# Comparing Unmet Service Needs Between Rural and Urban Family Caregivers of People Living with Alzheimer’s Disease and Related Dementias: A Multi‐Site Qualitative Study

**DOI:** 10.1002/alz.089591

**Published:** 2025-01-09

**Authors:** Christina E. Miyawaki, Angela McClellan, David Russell, Erin D. Bouldin

**Affiliations:** ^1^ University of Houston Graduate College of Social Work, Houston, TX USA; ^2^ Appalachian State University Department of Sociology, Boone, NC USA; ^3^ University of Utah School of Medicine, Salt Lake City, UT USA

## Abstract

**Background:**

The scarcity of resources and caregiving services, a higher prevalence of health conditions, and financial concerns in rural areas in the United States have been well‐documented. However, less research has compared experiences between caregivers of people with Alzheimer’s disease and related dementias (ADRD) in rural versus urban areas. This study sought to address this gap by identifying those unmet service needs, explore factors associated with service use, and propose tailored strategies for better serving the needs of both rural and urban caregivers.

**Methods:**

Using semi‐structured interviews guided by Andersen’s theory of health service use (1968), Taylor’s theory of dependent care (2001), and Roberto’s model of service use (2022), we interviewed 20 family caregivers of people with ADRD in rural areas of Western North Carolina and 18 caregivers within the urban setting of Houston, Texas. We compared their unmet service needs and contextual factors that facilitate their service use. We conducted a thematic analysis of interview data first with rural caregivers and developed a codebook. Using the same codebook, we analyzed the urban interview data while inductively adding new codes.

**Results:**

Rural caregivers were 56 years old (mean), working, White, and female. Urban caregivers were 63 years old, retired, White, Asian, or Black, and female. We found similar unmet service needs among rural and urban caregivers; however, the ways they approached and solved their challenges differed. Caregivers in rural areas wished for more information and caregiver support whereas urban caregivers looked for information they needed until they found the answers. Rural caregivers expressed guilt about using services because they felt they were limited and zero‐sum while urban caregivers shared available resources with others so that other caregivers could use them as well. Unmet service needs for urban caregivers included more culturally tailored services for people with ADRD in their ethnic‐specific languages and foods while rural caregivers’ cultural needs were not racially and ethnically specific but more place‐specific services.

**Conclusions:**

Increasing options for online and virtual support groups across the United States for rural caregivers and increasing culturally tailored service options would likely increase access and use for urban ADRD caregivers.

